# The Institutional Library

**Published:** 1920-05-01

**Authors:** 


### THE INSTITUTIONAL LIBRARY.

### THE OSLER SEPTUAGINT.

### A Tribute which has Become a Memorial.

When Sir William Osler was approaching his seventieth birthday it occurred to a band of his friends, colleagues, and pupils, both in England and in America, to offer him as a tribute of their affection and admiration, a collection of original scientific papers dedicated to him for the occasion of his birthday. The project was carried through, but before the papers could be published death had removed the beloved physician in whose honour they had been gathered together. The tribute of friendship has thus become a memorial; and a splendid memorial it is.* In two stately volumes the contributors, many of whose names are household words in medical circles on both sides of the Atlantic, offer of their best. Many of the articles are historical in purport; for was not Osler himself one of the keenest and most erudite medical historians of the day? Here, for instance, is an account, translated from the Italian, of the first recorded case of perforation of a duodenal ulcer (in the eighteenth century); there, a memoir by the grand old man of English-speaking medicine, Abraham Jacobi (now over ninety) of another fine old veteran, the late Sir Hermann Weber, who lived to be ninety-five.

Besides historical and biographical memoirs there are also a number of essays upon matters of medico-social politics; upon the teaching of medicine, for instance, by Sir William Hale White; on the future of the City hospitals in London, by Sir D'Arcy Power; on the problem of graduate medical study in London, by Professor Adami; and similar topics of institutional interest and educational importance. But the bulk of the contributions are of medical and scientific interest—including several from surgeons, by the way, on surgical topics, amongst which the contributions of the Mayos and of Matas may be mentioned. The essays deal with many different aspects of disease; some with problems which are only incidentally connected with disease at all, for instance, Professor Boycott's delightful excursus upon the "importance of being rather small." To summarise, even to catalogue, them is impossible. To have inspired so distinguished a body of men to the production of a work like this is reward enough for any scientist in his lifetime, and epitaph enough after his death. Osler was not only a great physician and a great man; above and beyond that he was greatly beloved, as these two volumes abundantly prove.

* *Contributions to Medical and Biological Research.* Dedicated to Sir William Osler, Bart., M.D., F.R.S., by his pupils and co-workers. (New York: Paul B. Hœber. 1919.)

**Lenzmann's Manual of Emergencies: Medical, Surgical, and Obstetric.** Based upon Lenzmann's "Emergencies in Medical Practice." By J. Snowman, M.D., M.R.C.P., Lond. (London: John Bale, Sons and Danielsson, Ltd. 1919. Pp. 324. Price 15s.)

There are few practitioners even in peace-time who have the time or the opportunity for keeping themselves abreast of the latest developments in all branches of their profession. More particularly does this apply to recent years, into which have been crowded new theories, fresh facts, revolutionised treatments, and new diseases, all happening at a time when the demands of the Army have so depleted the home ranks and so increased their labours as to leave them little time to study. It is opportune, therefore, that Dr. Snowman has produced his admirable compendium, based on Lenzmann's Manual, of the most common emergencies in medicine, surgery, and obstetrics. Each subject is treated very thoroughly, clearly, and tersely. The pathology is explained; the diagnosis simplified, and the physiological action of every drug described in such a manner as to enable the busy practitioner to acquire the latest teachings of standard authorities with a minimum of delay. The subjects are conveniently arranged in sections—respiratory, heart, nervous, gastro-intestinal, urinary, poisons, and midwifery, and with the list of contents and the index, the treatment for any emergency can be looked up in a few minutes.

A few illustrations will indicate the character of the work. In the respiratory section, "hæmoptysis" occupies eight pages, the causes being classified into two groups: (*a*) Changes in the lung tissue; (*b*) Changes in the pulmonary circulation. The principle examples of the first group are phthisis, gangrene, cirrhosis of lung, pneumonia, bronchiectasis and malignant disease. The second group includes mitral stenosis, infarction of the lung, arterio-sclerosis of the bronchial or pulmonary vessels. Then follows a description of the pathology of each, and a special paragraph on aneurism of the aorta. A page and a half is devoted to diagnosis and prognosis and the same to treatment. The aim of all treatment is to encourage the formation of a thrombus at the site of the hæmorrhage, and to secure its organisation, and the means to this end are clearly indicated. It is pointed out that the method of treating hæmoptysis by ergot, adrenalin, or other astringents is decidedly bad, as these drugs do not constrict the pulmonary vessels; they constrict other blood-vessels in the body with a consequent risk of an increase of pressure in the pulmonary vessels which encourages further hæmoptysis. Rational treatment requires a diminution of blood pressure in the pulmonary circulation, and the various means of bringing this about are described. Among dangerous emergencies in diseases of the nervous system the pathology of shock is particularly well described. In the section on nerve poisons the treatment of cocaine poisoning is thus described: "A 1 in 500 solution of tannin should be given at once, as it forms an insoluble compound with cocaine. The resulting tannate of cocaine can then be removed from the stomach by lavage or by an emetic." This advice would appear to be somewhat misleading; unless the stomach be very full the paralysing action of cocaine on the sensibility of its lining membrane is very rapid and an emetic is inert, an immediate copious draught of tea, if available, or of

**The Institutional Library**—(*continued*).

water, simultaneously with the hypodermic injection of apomorphine is a much more reliable method of saving life. There is very little, however, which is open to criticism in a work which is full of knowledge calculated to benefit suffering humanity and to aid the busy practitioner who is unable to stock his library with the latest editions on every subject or to find the time to read them.

**Diseases of the Throat, Nose, and Ear for Practitioners and Students.** By W. G. Porter, M.B., B.Sc., F.R.C.S.Ed. Third Edition, fully revised under the Editorship of A. Logan Turner, M.D. Ed., F.R.C.S. Ed. With a photogravure portrait of Major Porter and 79 illustrations, 44 of which are in colours. (Bristol: John Wright and Sons. 1919. Pp. 300. Price 12s. 6d. net.)

This book was first published in 1912, the second edition appeared in 1916, having been revised by Dr. McBride during the author's absence on active service. He was killed in action on June 8, 1917, and his old friends and former colleagues in Edinburgh have now undertaken a further revision and produced the third edition. There is not much to add to our previous notice of the work, as it has not been greatly altered and is, as before, an extremely good text-book of its size and class, written with care and judgment, and very well illustrated. Dr. J. Milne Dickie has added an excellent exposition of vestibular tests; Dr. J. T. Fraser has revised the sections on nerve-deafness, labyrinthine suppuration, otosclerosis and chronic catarrhal deafness; and Dr. Logan Turner, Dr. Douglas Guthrie, and Dr. W. T. Gardiner have revised other portions. It is one of the best small text-books on the subject, and may be heartily recommended to the student and practitioner.

**The Welfare of the Expectant Mother.** By Mary Scharlieb, G.B.E., etc. (Cassell, 5s. net.)

In *The Welfare of the Expectant Mother* Mrs. Scharlieb collects those wise and well-balanced words to which her lectures have accustomed us into a book which touches upon almost every topic included in this wide subject. The first three chapters deal with the hygiene and pathology of pregnancy and are followed by a chapter on the respective duties of doctor, midwife, maternity nurse, and health visitor. The sound common-sense which characterises the writer's work is noticeable in such a passage as that which says that in the homes of the poor the maternity nurse will see that the children are washed, fed, and sent to school, though these things are no part of her professional duties and add to her fatigue. That the industrial mother may be over-visited unless these various persons work in thorough harmony is a wholesome reminder, but we are glad to find that Mrs. Scharlieb has a good word for the health visitor and sympathy with difficulties of her rather uncharted work. The second part of the volume is occupied with the administrative care of the expectant mother. Mrs. Scharlieb puts briefly but forcibly the facts with regard to the loss of infant life through venereal disease and those of industrial employment, and here points out what is often forgotten, that ignorance and neglect of mothercraft is necessarily more serious among industrial mothers than in the leisured classes where paid workers can replace the mother to some extent. Therefore it becomes a matter of national concern. She is hopeful that better pay and increased leisure will lead the working man to provide more carefully for the needs of his family, a point of view which is not common, but may be justified, as education and more time spent in the home may draw out more thoughtful attention. The chapter on illegitimacy contains much that is important, especially in its strong plea for efficient teaching of the young, and the further plea for legislation which will put the onus of affiliation proceedings upon the State and not upon the betrayed girl. At the same time readers are reminded that not even consideration for the innocent and helpless child should blind us to the fact that its mother needs not only sympathy and help, but moral regeneration—a not unneeded warning. The last chapter, which deals with laws and regulations, discusses the burning questions of the supply of midwives and the present difficulties of their position. The book is one which, while instructive to professional workers, may with great advantage be read also by women interested in the whole subject, whether or not they are taking any part in social work.

**Change of Life: Its Difficulties and Dangers.** By Mary Scharlieb, G.B.E. (Scientific Press. 1919. Pp. 123. Price 1s. 3d. net.)

This is a small but valuable book explaining in language which any woman can understand the nature and the difficulties of, and the proper way to comport oneself towards the menopause, or "change of life," which normally affects every woman somewhere between forty and fifty years of age. Very few men (except doctors, and not all of them) realise the special trials which may attend this peculiar phenomenon, and a great many women quite misunderstand both their own symptoms and the non-comprehension of their masculine relatives. Both have much to learn from Mrs. Scharlieb's excellent exposition, for she speaks both as a physician and as a woman. There is, of course, a tendency among neurotic women, especially vain ones beginning to realise that the special charm of youth is no longer theirs and foolishly repining at the unpleasant revelation, to exaggerate their troubles, or to invent wholly imaginary ones. This type of woman will not find encouragement from Mrs. Scharlieb, but she will find, as other women will, much valuable counsel from following which she can derive nothing but benefit.

**Syphilis in Childhood.** By Leonard Findlay, M.D., D.Sc., F.R.F.P.S.G. (London: Henry Frowde; Hodder and Stoughton. 1919.)

The publications on the subject of venereal disease and its various manifestations have, since the end of the war, been more remarkable for the number than their universal excellence, but we unhesitatingly classify Dr. Findlay's work among those which should be placed in the library of every practitioner who wishes to have a comprehensive set of works on the subject. Both the clinical description and illustrations are excellent.

**Cunningham's Practical Anatomy.** Edited by Professor Arthur Robinson. Seventh edition. Vol. I. "Superior Extremity and Inferior Extremity"; Vol. II., "Thorax and Abdomen"; and Vol. III., "Head and Neck." (London: Henry Frowde, Hodder and Stoughton. Price 12s. 6d. net per vol.)

We are in receipt of Vol. I. and II. of this series, and in their modern and revised form bid fair to maintain the position of the "Small Cunningham" as the premier work to accompany practical anatomical dissection. Both new and old terminology are given, many new illustrations provided, and the whole brought thoroughly and efficiently up to date.

